# Built Environments and Cardiometabolic Morbidity and Mortality in Remote Indigenous Communities in the Northern Territory, Australia

**DOI:** 10.3390/ijerph17030769

**Published:** 2020-01-25

**Authors:** Camille Le Gal, Michael J. Dale, Margaret Cargo, Mark Daniel

**Affiliations:** 1 Institut de Santé Publique, d’Epidémiologie et de Développement, Université de Bordeaux, Bordeaux 33000, France; camille_legal@yahoo.fr; 2 Health Research Institute, University of Canberra, Bruce, ACT 2617, Australia; michael.dale@canberra.edu.au (M.J.D.); margaret.cargo@canberra.edu.au (M.C.); 3 Department of Medicine, St Vincent’s Hospital, The University of Melbourne, Fitzroy, VIC 3065, Australia

**Keywords:** epidemiology, built environment, Indigenous health, mortality, morbidity, cardiometabolic disease, remote

## Abstract

The health of Indigenous Australians is dramatically poorer than that of the non-Indigenous population. Amelioration of these differences has proven difficult. In part, this is attributable to a conceptualisation which approaches health disparities from the perspective of individual-level health behaviours, less so the environmental conditions that shape collective health behaviours. This ecological study investigated associations between the built environment and cardiometabolic mortality and morbidity in 123 remote Indigenous communities representing 104 Indigenous locations (ILOC) as defined by the Australian Bureau of Statistics. The presence of infrastructure and/or community buildings was used to create a cumulative exposure score (CES). Records of cardiometabolic-related deaths and health service interactions for the period 2010–2015 were sourced from government department records. A quasi-Poisson regression model was used to assess the associations between built environment “healthfulness” (CES, dichotomised) and cardiometabolic-related outcomes. Low relative to high CES was associated with greater rates of cardiometabolic-related morbidity for two of three morbidity measures (relative risk (RR) 2.41–2.54). Cardiometabolic-related mortality was markedly greater (RR 4.56, 95% confidence interval (CI), 1.74–11.93) for low-CES ILOCs. A lesser extent of “healthful” building types and infrastructure is associated with greater cardiometabolic-related morbidity and mortality in remote Indigenous locations. Attention to environments stands to improve remote Indigenous health.

## 1. Introduction

Despite government policy aimed at improving health outcomes for Aboriginal and Torres Strait Islander (hereafter, Indigenous) Australians [1], Indigenous communities continue to face significant challenges that yield substantial disparities in terms of cardiometabolic disease (CMD) [2]. Recent surveys indicate that Indigenous Australians are 3.3 times more likely to have diabetes compared to the non-Indigenous population [3], the age-standardised death rate for ischaemic heart disease is twice as high for the Indigenous population than for the non-Indigenous population [4], and Indigenous adults are 1.2 times more likely than non-Indigenous adults to be hypertensive [5].

Although the health disparity between Indigenous and non-Indigenous Australians is well documented by descriptive analyses, with studies providing information on the mechanisms linking individual risk factors to CMD in these populations [6,7], these mechanisms likely represent only a portion of the overall risk of CMD in the Indigenous Australian population. It is long acknowledged that individual risk factors are always intrinsically dependent upon the environmental, socio-political, and socio-cultural contexts that condition lifestyles within the population [8]. There is, however, a dearth of studies of the Indigenous Australian population that approach the Indigenous/non-Indigenous health disparity from a perspective inclusive of the environmental context. The relationship between the environment and Indigenous health is complex [9] and cannot be described solely in light of individual risk factors [2]. A broader conceptualisation is necessary, inclusive of the contribution of environmental factors to Indigenous health. Such an approach would allow for the heterogeneity in the environmental contexts to which Indigenous people are exposed, as well as heterogeneity in corresponding population compositions [2,10], to be acknowledged.

Focusing only on individual data fails to account for the complexity of environments and how, particularly for Indigenous Australians, variations in environments are both shaped by and determine spiritual, social, physical, material, economic, cultural, and political bonds to the land [9,11,12]. Central to this notion of environmental influences on Indigenous health is the ongoing challenge of providing quality, culturally appropriate housing in remote communities [13]—a challenge long acknowledged in the literature [14,15]. However, housing alone does not completely define the built environment of remote Indigenous communities. Other elements of the built environment vary by community and stand to influence the health of residents, in the same way that features of the built environment can influence health in non-remote contexts [16,17,18]. Non-housing-related built environment features of Indigenous communities are not frequently assessed with respect to their potential to impact on resident health outcomes. There is, therefore, a significant knowledge gap for the Indigenous population, regarding the impact of heterogeneity of the local built environment on heterogeneity in disease outcomes. The objective of this study was to determine the associations between the built environmental context and CMD-related morbidity and mortality in remote, predominantly Indigenous communities in the Northern Territory (NT) of the Commonwealth of Australia. We assessed associations between the relative “healthfulness” of the built environment and CMD-related morbidity and mortality.

## 2. Materials and Methods

### 2.1. Study Design

This study was part of the Environments and Remote Indigenous Cardiometabolic Health (EnRICH) project, a cross-sectional epidemiological study using aggregated geographic and community-level health outcome data. Ethics approval for the present study was obtained from the Human Research Ethics Committee (HREC) of University of South Australia (HREC Reference No. 31874), the Central Australian Human Research Ethics Committee (HREC-13-182), and the Human Research Ethics Committee of the Northern Territory Department of Health and Menzies School of Health Research (HREC 2013-2083). The purpose of this study was specifically to reach beyond the predominantly descriptive studies done thus far to evaluate inferential relationships between built environmental exposures and cardiometabolic outcomes for remote Indigenous communities throughout the NT.

### 2.2. Setting

Communities were included within this study if they met the following eligibility criteria: (1) they were located within the borders of the NT of Australia, (2) population size was 50 persons or more, (3) the proportion of Indigenous residents was 70% or more of the total community population, and (4) the community was defined as “remote” according to the Australian Standard Geographical Classification [19].

A total of 833 Indigenous communities located within the NT were identified though the Australian Government Indigenous Programs and Policy Location (AGIL) 2013 dataset. Of this total, 693 communities were excluded because their population was less than 50 persons, and 17 communities were excluded because the proportion of Aboriginal and Torres Strait Islanders was less than 70%. The resultant total of 123 AGIL-defined eligible communities were then matched to 104 Indigenous locations (ILOCs). ILOCs are the smallest resolution at which census data for the Indigenous population are available from the Australian Bureau of Statistics (ABS) [19]. An ILOC typically represents a small Indigenous community, although some ILOCs aggregate multiple very small and geographically proximal communities within a limited geographic area. Sociodemographic data, including *population size* (all persons), *proportion of Indigenous residents*, *median age* (Indigenous persons), and *gender ratio* (females to males), were extracted from the ABS 2011 Population and Housing Census [20], expressed at the level of the ILOC.

Of the 104 ILOCs, 13 contained more than one AGIL-defined community accounting for the “extra” 19 AGIL-based communities. Hence *n* = 91 AGILs were a 1:1 match with an ILOC. The unit of observation and analysis was the ILOC. Where multiple communities were present within an ILOC, community-level outcome and built environment exposure data were aggregated to create ILOC-level data.

### 2.3. Variables, Data Sources, and Measurement

#### 2.3.1. Outcome Data

For each community, CMD-related morbidity and mortality data were extracted from government registers, described below. All such observations were assigned to a specific community via the common “usual community of residence” record field. Under the rubric of CMD, this study includes hypertension, stroke, cardiovascular disease, and type-2 diabetes mellitus. Four distinct sets of outcomes were compiled for the period 1 January 2010 through 31 December 2015 for each community: (1) primary healthcare data reflecting CMD-related morbidity, obtained from the Primary Healthcare Collection of the NT Department of Health, were expressed as the count of CMD-related visits to NT Primary Healthcare wards; (2) inpatient admissions, reflecting CMD-related morbidity, obtained from the Inpatient Activity of the NT Department of Health, were expressed as the count of CMD-related inpatient admissions; (3) emergency department events reflecting CMD-related morbidity, obtained from the Emergency Department Data Collection of the NT Department of Health, were expressed as the count of CMD-related admissions recorded by emergency departments; (4) CMD-related mortality, obtained from the NT-wide mortality dataset of the Births, Deaths, and Marriages office (NT Department of the Attorney General and Justice), was expressed as the count of deaths deemed to be caused by CMD.

The four sets of outcome data did not have complete coverage across the 104 ILOCS within the study. The dataset with the highest level of coverage was the mortality dataset (*n* = 96). Coverage was lower for the other outcome datasets (*n* = 91 for inpatient admission, *n* = 79 for emergency department admissions, and *n* = 69 for primary healthcare visits). Lower coverage for the primary healthcare outcomes arose because the data source (NT Department of Health) does not capture data for non-governmental (community, not government-controlled) primary healthcare centres that operate in the NT.

All outcomes were analysed in relation to community-specific population denominators extracted from the ABS 2011 Census [20] and aggregated to and expressed at the ILOC level.

#### 2.3.2. Exposures Data

Built environmental exposure data were extracted from Serviced Land Availability Programme (SLAP) maps maintained by the NT Department of Lands, Planning, and the Environment. SLAP maps identify each building within a community and other community resources such as sports fields and infrastructure. Each building or other infrastructure element within each community was assigned to a category based its function, purpose, or status. These categories were “accommodation”, “aged care”, “child care”, “community”, “disused building”, “education”, “health”, “industry”, “infrastructure communication”, “infrastructure power”, “infrastructure sewage”, “infrastructure shelter”, “infrastructure toilet”, “infrastructure transport”, “infrastructure water”, “religion”, “retail”, “services”, “sport and recreation”, “storage”, “transport”, “unfinished”, “arena”, or “oval”. To assess the impact of the presence or absence of these buildings and associated services (i.e., the overall built environment context), each of these categories of buildings and infrastructure was classified into either the “healthful” or “unhealthful” category. This categorisation was conducted with reference to a pre-existing framework for operationalising place effects on health and focused upon the contribution of the building type to provide services and/or contribute to a healthy environment [21,22,23,24,25,26,27]. All categories of buildings and infrastructure were coded “healthful” except for “disused buildings” and “unfinished buildings”, which were coded “unhealthful”. The “industry” and “religion” building categories were removed based on the contradictory data in the literature that did not allow these variables to be defined as either healthful or unhealthful [28,29,30]. The presence or absence of buildings or infrastructure within each category was established for each community.

To characterise the built environment context of each community, a cumulative exposure score (CES) was created. For each “healthful” building type or infrastructure category present within a community, a value of 1 was assigned. For each “unhealthful” building of infrastructure category present within a community, a value of −1 was assigned. Overall exposure scores were created by summing all values for each community. The maximum possible CES score was thus +20, and the theoretical minimum was −2.

### 2.4. Statistical Method

Statistical analyses were performed using R Version 3.4.4 (R Foundation for Statistical Computing, Vienna, Austria) with statistical significance set at an alpha value of *p* = 0.05. Associations between built environmental exposures and CMD outcomes were assessed using a quasi-Poisson regression model [31]. ILOCs were dichotomised (based on a median split of the CES score) into high-CES (equal to 11 or more, indicative of a greater variety of healthful building available in the community) and low-CES (10 or less) categories. Analyses were then performed to compare the low-CES category relative to the high-CES category for the four sets of CMD-related outcomes, with *population size* used as the offset term in quasi-Poisson models to give greater weight to larger ILOCs. Potential cofounders adjusted for were *median age* and *gender ratio*. All quantitative continuous adjustment variables were modelled as a fractional polynomial function [32].

## 3. Results

### 3.1. Descriptive Data

For the 104 ILOCs included in the study, the mean population size was 370.3 (SD 416.3) persons, the average median age (years) of the Indigenous population corresponded to young adulthood, 23.0 years (SD 4.3), and the mean gender ratio was 1.1 females to males (SD 0.2) (see Table 1). Seventy-nine ILOCs (78.2%) lacked water infrastructure, 98 (97.0%) lacked sewerage infrastructure, and 62 (61.4%) lacked power infrastructure buildings. In addition, buildings dedicated to education were absent for 34 ILOCs (33.7%), and buildings for sport or recreation were absent in 66 ILOCs (65.3%). Approximately half the ILOCs within the sample did not have local access to retail or community buildings (48.5% and 43.6%, respectively). Given that the presence of one such resource within *any* of the communities within an ILOC representing multiple communities was sufficient to result in that ILOC being coded as “having” that resource, these data likely slightly underestimate the numbers of *communities* that lack access to these resources.

### 3.2. Main Results

Analyses of built environmental exposures expressed per the CES indicated a higher risk of CMD-related mortality and morbidity for ILOCs with low relative to high CES. These results were statistically significant for three of the four data sources (see Table 2). Moderate-to-large and statistically significant elevations in risk for low-CES ILOCs were observed for CMD-related inpatient admissions (relative risk (RR) 2.41, 95% confidence intervals (CI) 1.25–4.64), CMD-related emergency department admissions (RR 2.54, 95% CI 1.15–5.60), and CMD-related mortality (RR 4.56, 95% CI 1.74–11.93). The relationship between CES score and CMD-related primary healthcare visits was consistent in direction and magnitude with that observed in other morbidity data sources but failed to reach statistical significance (*p* = 0.079).

## 4. Discussion

This study assessed the associations between features of the built environment and cardiometabolic health in remote Indigenous communities in the Northern Territory of the Commonwealth of Australia. Using a collective score reflecting the variety of buildings and infrastructure present in the community, we investigated the relationships between built environment features of remote ILOCs and CMD-related morbidity and mortality. We observed that a lower extent of healthful built environment features was associated with higher ILOC-level rates of CMD-related morbidity and mortality. While these data are reported at the ILOC level, a high proportion (91 of 104, 87.5%) of these ILOCs represent a single community. The observed relationships are likely to hold at the community level also.

The direction of these relationships was consistent, independent of the outcome type assessed. The magnitude of these relationships varied but was substantially higher for CMD-related mortality data compared to CMD-related morbidity data. This may be a consequence of superior coverage of the mortality dataset as compared with the morbidity data. If this is so, it is possible that the healthful built environment–morbidity relationships observed herein are underestimated. However, it is probable that lack of some healthful built environment resources is inherently more impactful on the risk of CMD-related events resulting in mortality (i.e., lack of health and medical resources sufficient to address acute, life-threatening CMD-related events) than non-life threatening acute events or ongoing chronic conditions, which may account for the stronger relationship observed for the mortality data. Despite the primary healthcare outcomes not reaching statistical significance (likely due to reduced sample size related to the absence of data from community-controlled primary healthcare centres), the similarity between magnitudes of relative risk within the morbidity data (RR 2.41–2.94) clearly indicate a similarly consistent strength effect of a reduced extent of healthful built environment resources on CMD-related morbidity in remote, predominantly Indigenous ILOCs. Low levels of healthful built environment features within the ILOC corresponds to twice the likelihood of CMD-related morbidity. Similarly, low levels of healthful built environment features within the ILOC corresponds to more than four times the likelihood of CMD-related mortality.

These data are the first, to our knowledge, to examine the influence of community-level built environmental features of remote indigenous communities in northern Australia on CMD-related health outcomes. Given the disproportionately poorer cardiometabolic health experienced by Indigenous Australians [33,34], the role of the built environment in such communities should receive further scrutiny. It is well established that the availability [35] and condition [36,37] of housing, along with provision of functioning household amenities [38], impacts Indigenous health. Similarly, the primary determinants of housing condition in rural and remote communities have also been explored in detail [39,40]. Housing alone, however, is but a component of the built environment; thus, investigations of the influence of the built environment on health should move beyond the focus on housing-related health effects alone. For example, non-housing built environment features (i.e., women’s centres, aged care facilities) were previously demonstrated to be associated with significantly lesser odds of carer-reported gambling problems (odds ratio (OR) 0.46, 95% CI 0.27–0.79 and OR 0.59, 95% CI 0.39–0.97, respectively), which are in turn associated with child health outcomes [41]. Other environmental-level factors were recently shown to influence dietary quality in remote Indigenous communities in the Northern Territory [42]. Such evidence, along with the observations of this study, begin to elucidate the long-implicated [2,43] role of community-level environmental features on health in remote Indigenous communities.

These findings have implications for government policy regarding service provision to remote communities. Recent federal and territory government adoption of a “hub and spoke” model of resourcing, which centralises the provision of housing and other services to “growth towns” [44], is contraindicated (on health grounds) by our data. The effect of such policy is, invariably, a reduction in the variety of services (and the attendant built environment features) in remote communities, consequent to consolidation in central locations [44], and an expectation that residents outside central locations would either temporarily or permanently migrate to access services [45]. Such consolidation and service loss in smaller, remote towns is already evident in neighbouring Western Australia [44]. Our findings demonstrate the potential for deleterious second-order effects on resident health in communities with low built environment variety—most likely the “spokes” in the “hub and spoke” model. In addition to the effect of loss of services in situ, there is doubt as to the ability of the “hubs” in this model to adequately service “spokes” [45]. Far from representing a policy complementary to the federal government target of reducing disparity between Indigenous and non-Indigenous health [1], the current centralisation model may work at cross purposes to this objective.

We acknowledge several limitations and potential biases within this study. Data extracted for the primary healthcare dataset correspond to the number of visits or consultations relating to CMD and not to the number of diagnoses of CMD as is the case for other data. Findings from analyses of primary healthcare data should be interpreted with some caution, as the nature of observations is not consistent with other datasets. The classification of built environment features as healthful or unhealthful lacks the nuance to assign levels or degrees of (un)healthfulness to each category of infrastructure or building type and, thus, assumes an impact of equal magnitude for each category. This is relevant to the relationship between low built environment “healthfulness” and mortality, which may be sensitive to the lack of specific infrastructure associated with emergency care. The classification procedure for the built environment CES score resulted in the removal of building types whenever classification was uncertain, as was the case for “industry” and “religion” building types. As existing studies on this subject come mainly from countries with Western lifestyles with little focus on remote Indigenous populations, there is the possibility of misclassification bias. Any such bias, however, is likely to be random rather than systematic, thus biasing the result to the null.

Another possible source of bias is the instability of Indigenous populations [46], which are subject to frequent changes in magnitude. Data regarding the number of people in the community at the time of the study may not be completely representative or exhaustive, and the “usual” community of residence might not be accurate for all residents. Again, however, such variation is likely to appear at random within the sample and, thus, would bias the results towards the null. Finally, this study is ecological, in that it deals with relationships between environmental factors and community aggregated individual data, not individual data per se. Ecological studies present challenges in interpretation given widespread tendencies to assume that what holds for ecological units (in this case, ILOCs) will hold for the individual. The results of this study cannot be logically extrapolated to the level of the individual. Regardless of these considerations, this study is unique in the combination of the breadth of scope (123 communities within 104 ILOCs) and inferential attention to the relationship between the built environment context of remote Indigenous communities and Indigenous health outcomes.

## 5. Conclusions

This study investigated the impact of the built environment on CMD-related morbidity and mortality in remote, predominantly Indigenous ILOCs. We observed that the presence of a lesser variety in healthful building types within an ILOC was moderately to strongly associated with higher levels of CMD-related morbidity and mortality in these ILOCs. The built environment of remote, predominantly Indigenous communities would bear more comprehensive examination to explicate the associations between it and disease outcomes and, thus, resident health.

## Figures and Tables

**Table 1:** Descriptive characteristics of Indigenous locations (ILOCs).

Characteristic of ILOCs (*n* = 104)	Mean	Standard Deviation	Minimum	Maximum
Total number of persons	370.3	416.3	57	2293
Percent of Indigenous in the population (%)	92.6	5.2	75.8	100
Median age of Indigenous residents (years)	23.0	4.3	14	35
Gender ratio (number of females per male)	1.1	0.2	0.59	1.71

**Table 2:** Relative risk of cardiometabolic disease (CMD)-related mortality and morbidity, low versus high cumulative exposure score (CES). RR—relative risk; CI—confidence interval.

Morbidity and Mortality Outcome	RR	Lower and Upper 95% CI	*p*-Value
CMD-related primary healthcare visits	2.94	0.90–9.61	0.079
CMD-related inpatient admissions	2.41	1.25–4.64	**0.010**
CMD-related emergency department admissions	2.54	1.15–5.60	**0.024**
CMD-related mortality	4.56	1.74–11.93	**0.003**

Note: reference category = high CES score; *n* = 69 for primary healthcare visits, *n* = 89 for inpatient admissions, *n* = 77 for emergency department admissions, and *n* = 95 for mortality. **Bold** *p*-values are statistically significant at α = 0.05.
